# Impact of Structured High-Frequency Disturbances on Linear Identification of Lateral Vehicle Dynamics

**DOI:** 10.3390/s26144562

**Published:** 2026-07-18

**Authors:** György Istenes, Dániel Pup

**Affiliations:** Vehicle Industry Research Center, Széchenyi István University, Egyetem tér 1, 9026 Győr, Hungary; pupd@sze.hu

**Keywords:** lateral vehicle dynamics, linear system identification, structured disturbances, narrow-band noise, notch filtering

## Abstract

This paper investigates the influence of weak but structured high-frequency disturbances on the linear system identification of lateral vehicle dynamics using experimental measurement data. The analyzed dataset originates from previously conducted driver-in-the-loop experiments involving free-driving and slalom maneuvers. Frequency-domain analysis confirms that the dominant vehicle dynamics are concentrated below approximately 2–3 Hz, while a weak but persistent narrow-band disturbance around 10 Hz is consistently present in the steering signal. To investigate the influence of this disturbance on identification, different disturbance-handling strategies are compared, including notch filtering, low-pass filtering, ARX, IV-ARX, and ARMAX model structures. The comparison considers prediction performance, model complexity, identified dynamics, and robustness under different measurement conditions. The results show that increasing the deterministic model order is generally less effective than either targeted preprocessing or explicit noise modeling. When the disturbance is spectrally well separated from the relevant vehicle dynamics, notch filtering combined with a low-order ARX model provides the most effective solution. If preprocessing is not possible, ARMAX models achieve comparable performance by representing part of the disturbance through the noise model. IV-ARX models are used as a benchmark to verify that the main conclusions remain valid under possible closed-loop bias. Based on these findings, a practical engineering workflow is proposed for selecting appropriate disturbance-handling strategies according to the spectral characteristics of the measured signals. The proposed methodology provides guidance for robust control-oriented identification of lateral vehicle dynamics using realistic measurement data.

## 1. Introduction

Understanding and modeling the lateral dynamics of road vehicles are essential for the development of driver assistance systems, vehicle stability control, and autonomous driving functionalities. Linear system identification methods play a key role in this field due to their simplicity, interpretability, and direct applicability in control-oriented modeling frameworks [1].

A fundamental observation in vehicle dynamics research is that the dominant behavior of lateral motion is concentrated in the low-frequency range. Frequency-domain analyses have shown that steering-induced responses are mainly significant below approximately 2–3 Hz, where the system exhibits predictable and nearly linear characteristics [2,3,4]. This has led to the widespread use of low-order linear models to represent lateral vehicle dynamics, particularly in controller design and validation.

The validity of this assumption has also been confirmed in experimental studies. Sensitivity analyses of the yaw rate and lateral acceleration responses indicate that vehicle behavior is most consistent and controllable in the low-frequency domain, while higher-frequency components tend to be dominated by noise, nonlinearities and unmodeled dynamics [5,6].

### 1.1. Vehicle Dynamics Identification and Measurement Disturbances

Real measurement data often contain structured disturbances that cannot be represented as simple white noise. These disturbances may originate from sensor characteristics, mechanical vibrations, or signal-processing artifacts, and frequently appear as narrow-band components in the frequency domain. Even when their energy is relatively small, their deterministic or quasi-periodic nature may affect parameter estimation by introducing spurious dynamics or distorting residual properties [1].

In the dataset analyzed in this study, a weak but persistent narrow-band component is observed around 10 Hz. Since the dominant lateral vehicle dynamics are concentrated below approximately 2–3 Hz, while the disturbance appears consistently under different maneuver types and vehicle speeds, it is treated as a structured narrow-band disturbance for the purposes of the present identification study. Throughout this paper, the term high-frequency refers to spectral components located outside the frequency range containing the dominant lateral vehicle dynamics, rather than to high frequencies in an absolute signal-processing sense. Although its energy is considerably lower than that of the dominant vehicle dynamics, its presence raises an important methodological question: should such a component be removed during preprocessing, represented explicitly by the identification model, or simply ignored?

Possible sources of this disturbance include sensor-related effects, communication and sampling artifacts associated with automotive data acquisition systems, and structural vibrations of steering or chassis components commonly encountered in vehicle NVH phenomena [7,8,9,10]. The objective of the present work is not to identify the physical origin of the disturbance, but rather to evaluate its influence on system identification and to determine the most appropriate strategy for handling it.

This problem is particularly relevant for black-box identification methods such as ARX (autoregressive with exogenous input) and ARMAX (autoregressive moving average with exogenous input). While ARX models tend to incorporate structured disturbances into the deterministic dynamics, ARMAX models can partially represent them through an explicit noise model. In addition, because the analyzed measurements were obtained under driver-in-the-loop conditions, possible closed-loop bias must also be considered. An instrumental-variable ARX (IV-ARX) estimator is included as a benchmark with reduced sensitivity to regressor-disturbance correlation.

### 1.2. Related Work

Diversi et al. investigated the identification of ARX and ARARX models in the presence of noisy input and output measurements [11]. The authors showed that conventional least-squares estimation may produce biased results under such conditions and proposed a dedicated identification procedure to address the errors-in-variables problem. Their work focuses primarily on reducing parameter estimation bias.

Yu et al. studied the identification of ARMA systems in the presence of quantized output measurements and colored noise [12]. By employing explicit disturbance modeling based on the Box–Jenkins framework, they demonstrated that appropriate noise modeling can significantly improve parameter estimation accuracy. Their results highlight the importance of separating deterministic system dynamics from disturbance dynamics.

Escobar et al. presented a comprehensive review of ARMAX model identification under non-Gaussian and correlated noise conditions [13]. Their work discusses whitening procedures, explicit noise modeling, and robust estimation techniques, providing a theoretical foundation for the improved robustness of ARMAX models observed in the present study.

### 1.3. Research Gap and Contributions

Although the effects of measurement noise and explicit disturbance modeling have been extensively investigated, comparatively little attention has been devoted to weak but persistent narrow-band disturbances that lie outside the frequency range of the relevant vehicle dynamics but remain embedded in real driver-excited measurement data. In practice, it is often unclear whether such disturbances should be removed through preprocessing, represented explicitly by the identification model, or simply tolerated.

The objective of this paper is to investigate the influence of such structured high-frequency disturbances on the linear identification of lateral vehicle dynamics using previously acquired experimental data [2]. Different preprocessing strategies, including notch filtering and low-pass filtering, are systematically compared together with ARX, IV-ARX, and ARMAX identification methods [14,15]. The IV-ARX models are included as a benchmark for assessing the robustness of the conclusions with respect to possible closed-loop estimation bias arising from driver-in-the-loop measurements.

Rather than identifying a universally optimal identification method, the aim of the study is to determine under which conditions each disturbance-handling strategy is most appropriate. The paper compares deterministic and stochastic disturbance-mitigation approaches in terms of prediction performance, model complexity, pole locations, and physical interpretability. Based on the obtained results, a practical engineering workflow is proposed for selecting preprocessing and identification strategies according to the spectral characteristics of the measured signals. The proposed methodology provides guidance for robust control-oriented vehicle dynamics identification using real measurement data obtained under realistic operating conditions.

## 2. Materials and Methods

The measurement campaign used in this study was carried out previously as part of a dedicated experimental investigation of lateral vehicle dynamics. Since the complete vehicle platform, sensor integration, and data acquisition architecture have already been described in detail in our earlier work, only the information directly relevant to the present study is summarized here. For a complete description of the experimental platform, including the drive-by-wire system and the full measurement framework, the reader is referred to [2].

All preprocessing, spectral analysis and system identification procedures were performed in MATLAB R2025b using the System Identification Toolbox.

### 2.1. Test Vehicle and Measurement System

The measurements were performed using a Lexus RX450h test vehicle modified for research purposes. The vehicle was equipped with an AutonomousStuff PACMod 3.0 drive-by-wire platform and an experimental data acquisition framework, enabling both manual and automated test scenarios. In the present study, only manually driven measurements were considered [16,17].

The signals used for identification were obtained from the vehicle’s onboard sensor network through the CAN bus [18,19]. The steering input was measured by the steering column angle sensor and converted to the equivalent front wheel steering angle. The lateral acceleration was acquired from the vehicle’s built-in inertial sensing unit through the electronic stability control signal chain and recorded from the CAN bus without additional filtering in the identification framework. These two signals constitute the input and output of the identified black-box model.

### 2.2. Test Conditions and Driving Maneuvers

The measurements were conducted on the high-speed handling section of the ZalaZONE proving ground in Hungary, which provides a controlled environment for repeatable lateral dynamic testing [2]. All measurements were taken on dry asphalt using Michelin all-season tires. The ambient temperature during the tests was approximately 7 °C.

Two main driving scenarios were considered:

- Free-driving tests, in which the vehicle completed laps on the test track at nearly constant speed, while the steering excitation was generated by the driver through natural steering corrections;
- Slalom tests, in which the driver guided the vehicle through a cone-defined path, producing a stronger and more periodic lateral excitation.

Free-driving measurements were carried out at nominal speeds of 50 and $65\;\frac{\mathrm{km}}{h}$. Slalom measurements were conducted at nominal speeds of 30, 50, and $70\;\frac{\mathrm{km}}{h}$. In the slalom tests, the cone spacing was adjusted according to the test speed in order to maintain a comparable maneuver severity. The cones were placed at 12 m spacing for the $30\;\frac{\mathrm{km}}{h}$ tests, 18 m for the $50\;\frac{\mathrm{km}}{h}$ tests, and 24 m for the $70\;\frac{\mathrm{km}}{h}$ tests.

In all cases, the actual vehicle speed remained close to the target value, with moderate deviations caused by driver behavior and track conditions. These maneuvers were selected because they provide representative low-frequency lateral excitation under realistic driver-in-the-loop operating conditions, while the slalom tests also introduce a more repeatable and structured steering excitation than the free-driving case.

### 2.3. Measured Signals and Sampling

The identification analysis presented in this paper is based on the measured steering angle and lateral acceleration signals. The equivalent steering angle of the front wheel was treated as the system input, while the lateral acceleration was considered the system output describing the lateral vehicle response. All signals were sampled at a frequency of 30 Hz. This sampling rate provides sufficient temporal resolution for the dominant low-frequency steering-induced responses while still preserving the weakly structured high-frequency disturbance observed in the measured data. As shown later, this disturbance appears as a narrow-band spectral component around 10 Hz and plays a central role in the identification problem investigated in this paper. The selected dataset provides an appropriate basis for evaluating the influence of structured high-frequency disturbances on linear system identification under realistic driving conditions.

## 3. Signal Analysis

This section presents the characteristics of the time and frequency domains of the measured steering angle and lateral acceleration signals used for system identification. The analysis has two objectives. First, it identifies the frequency range containing the dominant vehicle dynamics. Second, it determines whether structured disturbances are present that may affect the parameter estimation process.

### 3.1. Time-Domain Characteristics

**Figure 1:**
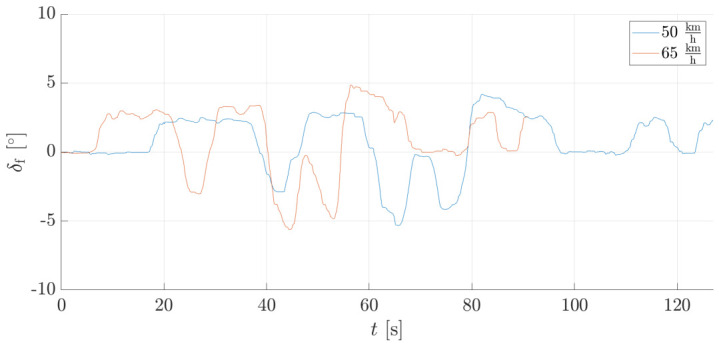
Steering angle ($\delta_{f}$) during the free-driving test.

**Figure 2:**
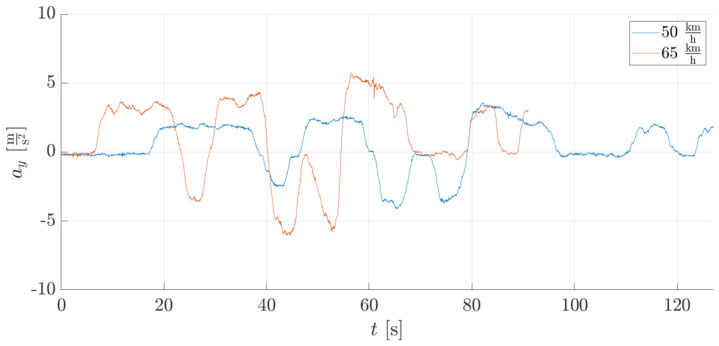
Lateral acceleration ($a_{y}$) during the free-driving test.

In free-driving measurements, the steering signal is characterized by relatively slow and irregular driver corrections required to maintain the desired trajectory. As a result, the lateral acceleration exhibits smooth transient responses with moderate amplitudes. At the higher test speed (65 $\frac{\mathrm{km}}{h}$), both the steering corrections and the corresponding lateral acceleration amplitudes increase compared to the 50 $\frac{\mathrm{km}}{h}$ case, indicating stronger lateral excitation at higher vehicle speed. One of the measurements obtained during the free-driving tests is shown in the time domain in Figure 1 and Figure 2.

**Figure 3:**
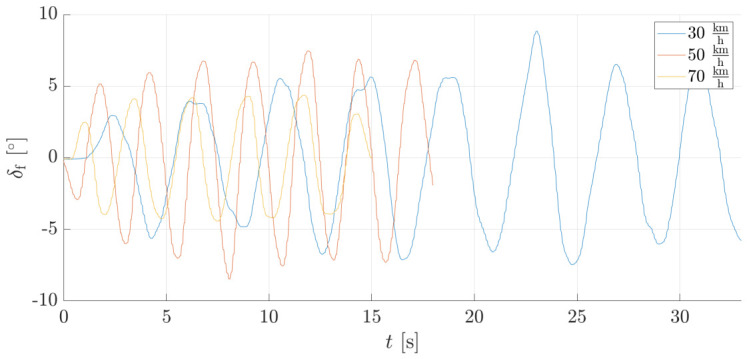
Steering angle ($\delta_{f}$) during the slalom test.

**Figure 4:**
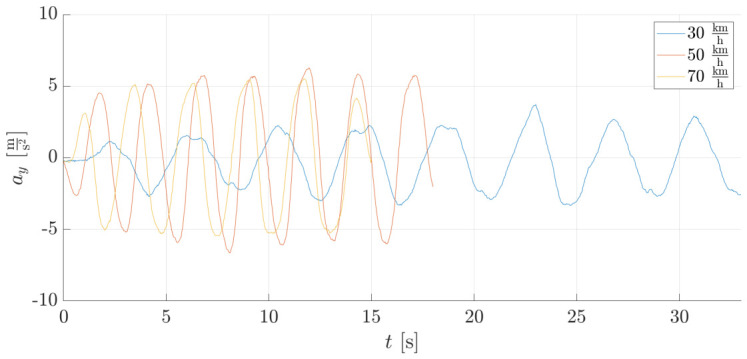
Lateral acceleration ($a_{y}$) during the slalom test.

In the slalom measurements, the steering input is quasi-periodic and significantly more structured due to the cone-defined maneuver. The resulting lateral acceleration closely follows the steering excitation and exhibits larger amplitudes than in free-driving. The response amplitude increases with vehicle speed, with the strongest excitation observed in the $50\;\frac{\mathrm{km}}{h}$ and $70\;\frac{\mathrm{km}}{h}$ cases. Despite the stronger excitation, the signals remain smooth in time, without visible oscillatory artifacts at higher frequencies. In these tests, the cone spacing was increased with vehicle speed, which partly compensates for the higher speed by increasing the maneuver radius. Nevertheless, the lateral acceleration remains speed-dependent, since for a given path curvature, it scales approximately with $\frac{v^{2}}{R}$. This explains why the steering angle amplitudes do not increase monotonically with speed, while the lateral acceleration response remains stronger in the higher-speed cases. One of the measurements obtained during the slalom tests is shown in the time domain in Figure 3 and Figure 4.

### 3.2. Frequency-Domain Analysis

The frequency-domain characteristics of the measured signals were investigated using the auto power spectral density (APSD). Frequency-domain analysis is used not only to characterize the measured signals but also to guide the subsequent identification strategy.

**Figure 5:**
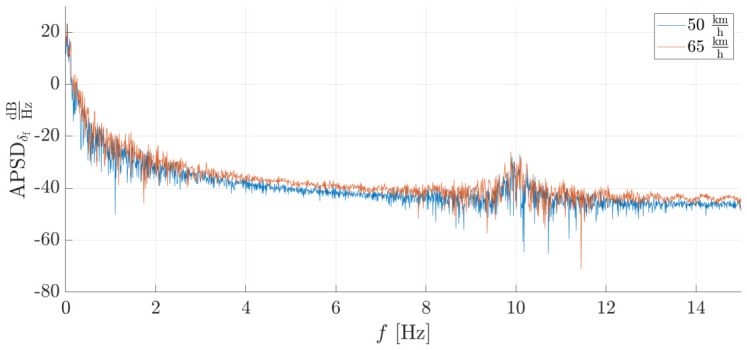
Steering angle ($\delta_{f}$) APSD during the free-driving test.

**Figure 6:**
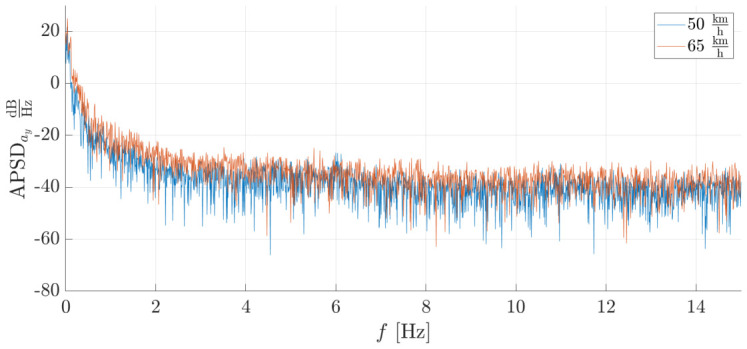
Lateral acceleration ($a_{y}$) APSD during the free-driving test.

In free-driving tests, the spectra exhibit a broadband low-frequency content associated with steering corrections and vehicle path-following behavior. The amplitude of spectral energy decreases rapidly with increasing frequency. This behavior is consistent with the expected low-pass characteristics of the lateral vehicle dynamics. One of the measurements obtained during the free-driving tests is shown in the frequency domain in Figure 5 and Figure 6.

**Figure 7:**
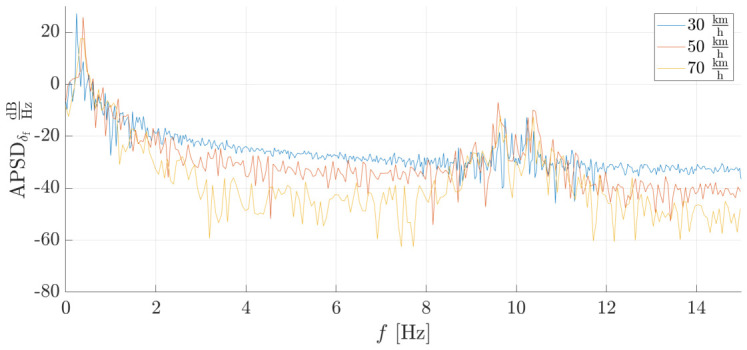
Steering angle ($\delta_{f}$) APSD during the slalom test.

**Figure 8:**
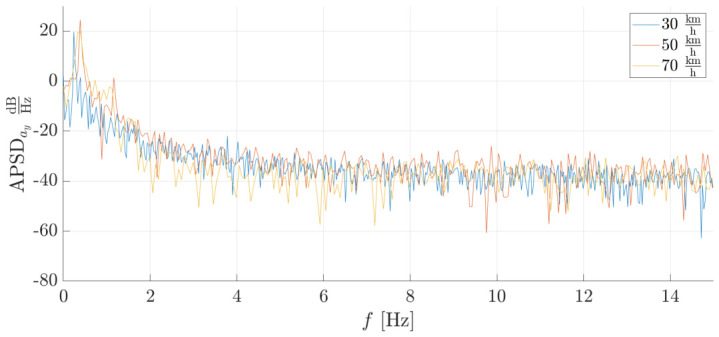
Lateral acceleration ($a_{y}$) APSD during the slalom test.

The slalom measurements show a more pronounced spectral structure due to the periodic nature of the maneuver. A dominant excitation peak is visible in the low-frequency range, corresponding to the steering rhythm imposed by the driver and the slalom geometry. The peak shifts slightly depending on the test speed, but remains clearly below the frequency range associated with measurement disturbances. One of the measurements obtained during the slalom tests is shown in frequency domain in Figure 7 and Figure 8.

The lateral acceleration spectra closely follow the low-frequency content of the steering input spectra, indicating strong dynamic coupling between the measured input and output variables. This confirms that the selected input–output pair is appropriate for the identification of control-oriented systems. In general, the frequency-domain analysis supports the use of low-order linear models.

### 3.3. Analysis of Structured Disturbances

Although dominant dynamics are concentrated in the low-frequency domain, APSD plots reveal a weak but clearly identifiable narrow-band spectral component around approximately 10 Hz. This peak is consistently present in the steering angle signal in all investigated scenarios, including both free-driving and slalom measurements. The disturbance is particularly visible in the steering angle spectra, where it appears as a distinct narrow-band peak approximately 30–50 dB below the dominant low-frequency content.

Although the energy of this component is relatively low, its narrow-band and deterministic nature make it potentially relevant for black-box system identification. In particular, ARX models may absorb this disturbance into the estimated system poles and zeros, while ARMAX models may partially represent it through the noise model. In both cases, the presence of such a structured disturbance can lead to biased parameter estimates, degraded residual whiteness, or unnecessary model complexity.

## 4. Identification Problem

In this section, the system identification task is formulated based on the measured data previously presented. The goal is to establish a linear input–output relationship between steering angle and vehicle lateral acceleration using black-box identification methods. The steering angle is considered the input of the system, while the lateral acceleration represents the output of the system. Identification is performed under real driving conditions, where the excitation is generated by a human driver, which introduces additional challenges compared to ideal open-loop experiments. The measurements also contain the structured narrow-band disturbance identified in Section 3.3, whose influence on identification is investigated in the following sections.

### 4.1. Linear Model Structure

For the identification of the lateral dynamics, discrete-time linear models are considered. In particular, ARX, IV-ARX, and ARMAX model structures are applied. These model classes are widely used in control-oriented system identification due to their relatively simple parametrization and their ability to represent linear dynamic relationships based on measured input–output data [1].

The ARX model can be written in the following form:

(1) $$A\left(q\right)y\left(t\right)=B\left(q\right)u\left(t-n_{k}\right)+e\left(t\right),$$

where y(t) is the output (lateral acceleration), u(t) is the input (steering angle), e(t) is a white noise disturbance, and *q* is the backward shift operator. Polynomials A(q) and B(q) are defined as follows:

(2) $$A\left(q\right)=1+a_{1}q^{-1}+\cdots +a_{n_{a}}q^{-n_{a}},$$

(3) $$B\left(q\right)=b_{1}q^{-1}+\cdots +b_{n_{b}}q^{-n_{b}},$$

where $n_{a}$ and $n_{b}$ are the orders of the polynomials A(q) and B(q), respectively.

The same ARX model structure is also estimated using an instrumental-variable approach. In this case, the model form remains identical to the ARX structure above, but the parameter estimation is modified in order to reduce the bias caused by correlation between the regressors and the disturbance. The IV-ARX models are estimated using the iv4 routine of MATLAB’s System Identification Toolbox, which implements a four-stage instrumental-variable algorithm [1]. In the following, these models are referred to as IV-ARX models.

The ARMAX model extends the ARX structure by explicitly modeling the disturbance dynamics:

(4) $$A\left(q\right)y\left(t\right)=B\left(q\right)u\left(t-n_{k}\right)+C\left(q\right)e\left(t\right),$$

where C(q) is a polynomial describing the noise model:

(5) $$C\left(q\right)=1+c_{1}q^{-1}+\cdots +c_{n_{c}}q^{-n_{c}},$$

where $n_{c}$ denotes the order of the polynomial C(q).

The ARMAX structure allows for a more flexible representation of disturbances compared to ARX, which is particularly important when the noise is not purely white. This makes ARMAX models more suitable for real measurement data where structured disturbances may be present.

### 4.2. Closed-Loop Effect

Unlike laboratory experiments with designed excitation signals, the measurements used in this study were obtained under driver-excited conditions. In this case, the driver continuously adjusts the steering input based on the observed vehicle response, effectively forming a feedback loop between the input and the output. As a consequence, the input signal u(t) cannot be assumed to be fully independent of the disturbance term e(t), which violates a key assumption of classical open-loop least-squares identification methods. The closed-loop identification problem is known to introduce bias in parameter estimation, especially for simple model structures such as ARX [20,21]. In practical terms, this means that part of the disturbance acting on the system may become correlated with the input signal through the driver’s reaction, which complicates the separation of the vehicle dynamics and the disturbance effects.

A rigorous treatment of closed-loop identification would require dedicated identification methods and a more detailed analysis of the driver–vehicle feedback mechanism. Such an investigation is beyond the scope of the present paper. Nevertheless, in order to reduce the sensitivity of the identified model to possible closed-loop bias, an instrumental-variable ARX benchmark is also included in the analysis. The IV-ARX estimator is less sensitive to regressor-disturbance correlation than standard least-squares ARX estimation, because it replaces the direct least-squares regression with an instrumental-variable procedure [1]. For this reason, in addition to the conventional ARX and ARMAX models, IV-ARX-based models are also evaluated. Their role is not to provide a complete closed-loop identification solution, but to assess whether the main conclusions regarding the influence of the structured disturbance remain consistent when a closed-loop-bias-mitigating estimator is used.

## 5. Noise Control Strategies

Two preprocessing strategies are investigated: targeted notch filtering and broadband low-pass filtering. The notch filter selectively removes the 10 Hz disturbance, whereas the low-pass filter suppresses both the disturbance and broadband high-frequency noise. The impact of these preprocessing approaches on system identification is investigated in the following sections. The objective of the preprocessing is not to remove a dominant component of the measured signal, but to suppress a structured disturbance that lies outside the frequency range of the relevant vehicle dynamics and may therefore bias the identification process.

### 5.1. Notch Filtering

To suppress the identified narrow-band disturbance, a second-order notch filter centered at 10 Hz is applied to the input signals. The filter is designed with a quality factor of Q=10, resulting in a relatively narrow attenuation band.

**Figure 9:**
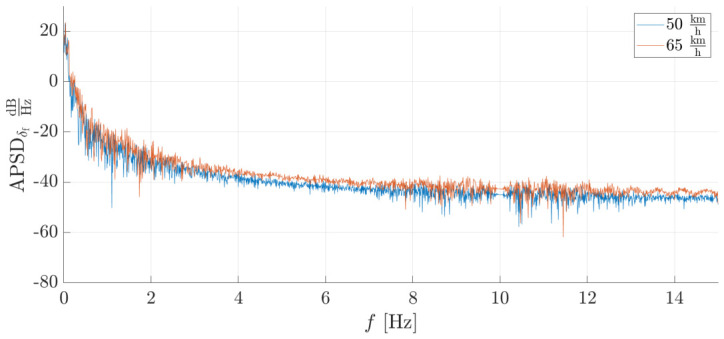
Notch filtering of steering angle ($\delta_{f}$) APSD during the free-driving test.

**Figure 10:**
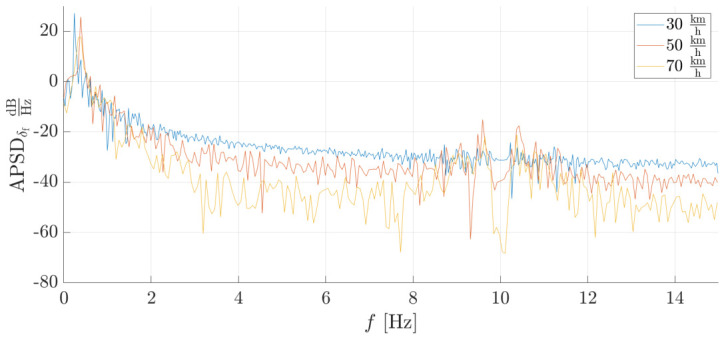
Notch filtering of steering angle ($\delta_{f}$) APSD during the slalom test.

As shown in Figure 9 and Figure 10, the notch filter effectively attenuates the spectral peak around 10 Hz while leaving the low-frequency content of the signals largely unaffected. The time-domain signals remain visually almost unchanged, indicating that the dominant vehicle dynamics are preserved.

This confirms that the disturbance is well localized in the frequency domain and can be selectively removed without significant distortion of the underlying system behavior. Similar behavior was observed for all measurement cases.

### 5.2. Low-Pass Filtering

As an alternative approach, low-pass filtering is applied to remove high-frequency components from signals. The filter is designed with a pass-band edge at 4 Hz and a stop-band edge at 6 Hz, with a pass-band ripple of 0.5 dB and a stop-band attenuation of 50 dB.

**Figure 11:**
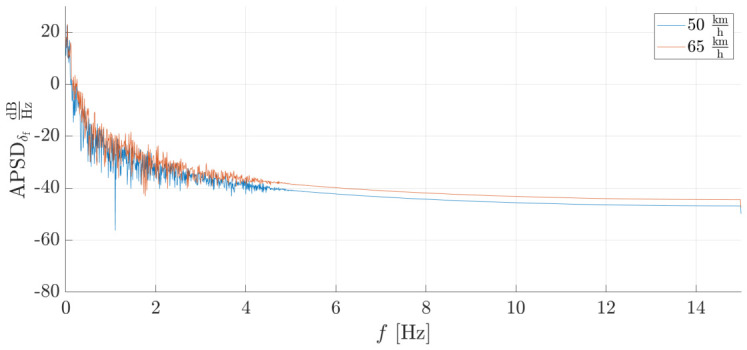
Low-pass filtering of steering angle ($\delta_{f}$) APSD during the free-driving test.

**Figure 12:**
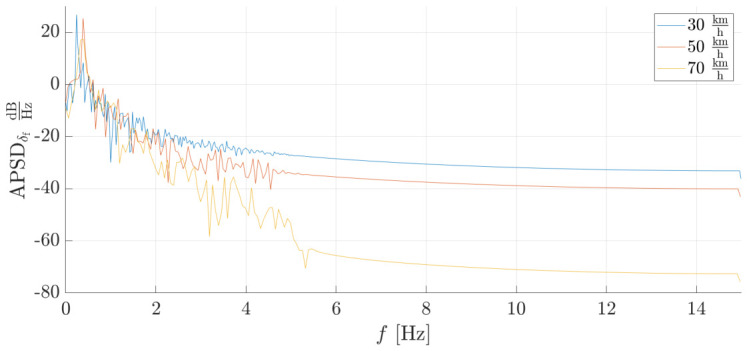
Low-pass filtering of steering angle ($\delta_{f}$) APSD during the slalom test.

Figure 11 and Figure 12 show that the low-pass filter effectively removes both the 10 Hz disturbance and the broadband high-frequency noise. Compared to the notch-filtered case, the resulting spectra are significantly smoother, and the high-frequency content is strongly attenuated. However, in contrast to notch filtering, low-pass filtering also affects the transition region of the signal spectrum. While the main dynamics are still preserved, some attenuation of higher-frequency dynamic components may occur. This effect is particularly visible in the frequency domain, where the spectral roll-off becomes significantly steeper.

## 6. Model Identification

The analysis focuses on the comparison of ARX, IV-ARX, and ARMAX model structures under different preprocessing conditions, including raw, notch-filtered, and low-pass-filtered measurement data. The identification results obtained from downsampled datasets are also evaluated as a reference investigation. The aim is not only to improve the numerical fit of the identified models, but also to evaluate their robustness, dominant pole behavior, and dynamical interpretability under realistic measurement conditions. Particular attention is devoted to the influence of the weak but structured high-frequency disturbance identified around 10 Hz, and to the question of whether such disturbances should be handled through preprocessing or through increased model complexity. Low-pass filtering is expected to provide a robust preprocessing strategy by suppressing physically irrelevant high-frequency components while preserving the essential system behavior. The downsampled datasets serve mainly as a complementary reference case, confirming the low-frequency nature of the relevant dynamics.

To enable a systematic comparison of the identified models obtained under different preprocessing conditions and model structures, several quantitative and qualitative evaluation metrics are considered. The selected criteria aim to assess not only the numerical fitting accuracy of the models but also their statistical robustness and physical interpretability. The following evaluation metrics are used throughout the identification analysis:

- FPE (final prediction error),
- BIC (Bayesian information criterion),
- One-step-ahead prediction fit,
- Dominant pole analysis.

### 6.1. Identification Cases and Preprocessing Overview

To evaluate the influence of structured high-frequency disturbances on linear system identification, several preprocessing strategies and identification scenarios were investigated. The analyzed datasets were derived from the same measured steering angle and lateral acceleration signals introduced previously, while different signal conditioning methods were applied prior to model estimation.

The ARX and ARMAX structures represent two widely used prediction-error model classes, while the IV-ARX approach was included to provide a benchmark that is less sensitive to possible closed-loop bias caused by the measurement configuration. All model types were identified under multiple preprocessing conditions in order to evaluate the sensitivity of the identified dynamics to structured disturbances and noise suppression techniques. Four main identification cases were considered:

- Raw measurement data sampled at 30 Hz,
- Notch-filtered signals sampled at 30 Hz,
- Low-pass filtered signals sampled at 30 Hz,
- Low-pass filtered and downsampled signals sampled at 10 Hz.

The first three cases were evaluated at the original sampling rate in order to compare the effect of different disturbance-mitigation strategies without changing the discrete-time representation of the identified system. In addition to the 30 Hz datasets, an additional reference investigation was performed using downsampled signals. A 10 Hz sampling rate still satisfies the Nyquist criterion with sufficient margin. Prior to downsampling, low-pass filtering was applied in order to suppress frequency components above the new Nyquist frequency and to avoid aliasing effects. The purpose of this investigation is not to propose downsampling as a primary disturbance-suppression technique, but rather to evaluate how the reduction in the effective bandwidth influences the identification problem and the resulting model structures. The filter parameters are the same as those described in the low-pass filtering section, except that the stopband frequency is set to 5 Hz. Since the discrete-time representation of the identified system depends on the sampling frequency, the downsampled datasets are not considered directly equivalent to the original 30 Hz cases.

The comparison of the identified models therefore serves two purposes. On the one hand, it reveals how different preprocessing strategies influence the robustness and interpretability of ARX- and ARMAX-type models in the presence of structured disturbances. On the other hand, the IV-ARX results provide an additional reference for assessing whether the main conclusions remain consistent when an estimation method with reduced sensitivity to regressor-disturbance correlation is used.

### 6.2. Model Order Selection

The selection of an appropriate model order is an important aspect of black-box system identification. In this study, the objective was not only to maximize the accuracy of the prediction but also to obtain parsimonious and physically interpretable models of the dominant lateral vehicle dynamics [1]. Since the dominant lateral behavior of road vehicles is commonly represented by low-order bicycle models and the spectral analysis revealed a single dominant low-frequency dynamical region, low-order ARX-, IV-ARX-, and ARMAX-type structures with gradually increasing polynomial orders were systematically investigated [3].

The selection of the model order was performed using the raw datasets, which preserve the full characteristics of the structured disturbance considered in this study. This choice makes it possible to evaluate not only the nominal predictive capability of the candidate model structures, but also their sensitivity to the disturbance under the most challenging identification conditions. The models were evaluated using the criteria introduced previously, namely FPE, BIC, one-step-ahead prediction fit, and dominant pole behavior. For ARX and IV-ARX models, the investigated structures are characterized by polynomial orders together with the input–output delay $\left(n_{a},n_{b},n_{k}\right)$, while ARMAX models also include the order of the noise model ($n_{c}$). The IV-ARX models retain the same deterministic model structure as the ARX models, but the parameters are estimated by an instrumental-variable procedure. Consequently, the comparison of the ARX and IV-ARX models allows the influence of the estimation method to be assessed separately from the order of the deterministic model. The results are summarized in Table 1, where $n_{k}=1$ and $n_{a}=n_{b}=n_{c}=\mathrm{order}$ for all cases. The reported values represent the averages obtained from repeated measurements, with two repetitions for the free-driving tests and three repetitions for the slalom tests.

The results indicate that increasing the model order generally improves prediction performance; however, the improvement rapidly diminishes beyond second-order models. This trend is observed consistently for the ARX and ARMAX structures, whereas higher-order IV-ARX models do not necessarily provide better performance under the investigated driver-in-the-loop conditions, likely due to the increased sensitivity of instrumental-variable estimation to limited excitation, measurement noise, and numerical conditioning. In several cases, second-order ARMAX models achieve prediction performance comparable to substantially higher-order ARX models, demonstrating that explicit noise modeling can effectively compensate for increased deterministic model complexity. The IV-ARX results also provide a useful benchmark for assessing the sensitivity of the conclusions to possible closed-loop bias. Although IV-ARX models do not consistently improve prediction performance, they confirm that the main observations regarding preprocessing and explicit disturbance modeling remain unchanged when an estimator with reduced closed-loop sensitivity is employed.

Based on these observations, second-order models were selected for the subsequent analysis. They provide an appropriate balance between prediction accuracy, physical interpretability, and model parsimony [22] while allowing a transparent comparison of preprocessing strategies and identification methods without introducing unnecessary model complexity.

### 6.3. Preliminary Assessment of Speed Dependence Using Raw Data

Before evaluating the different preprocessing strategies, the speed dependence of the identified model parameters is examined using the raw measurements. This provides a baseline that separates speed-related changes in the vehicle dynamics from those introduced by preprocessing or resampling. Second-order models selected in the previous subsection were identified from the raw datasets at different speeds, and the resulting parameters were compared across repeated measurements. The corresponding mean values and deviations are summarized in Table 2.

The identified parameters exhibit a measurable dependence on vehicle speed; however, the observed trends vary with both the model structure and the driving maneuver. For the ARX models, the most pronounced changes occur in the input polynomial coefficients ($b_{1}$ and $b_{2}$), indicating that the steering-to-lateral-acceleration relationship is not strictly speed-invariant within the investigated range. The autoregressive coefficients show weaker and less consistent variations. The IV-ARX models provide less consistent results, with larger parameter deviations and no clear monotonic trends. This behavior reflects the higher sensitivity of instrumental-variable estimation to the investigated measurement conditions and supports the use of IV-ARX primarily as a closed-loop benchmark rather than for interpreting speed-dependent parameter variations. The ARMAX models exhibit the most consistent parameter evolution across the investigated speeds while maintaining relatively small deviations. This suggests that explicit disturbance modeling improves the robustness of the identified deterministic parameters.

Overall, the raw-data comparison confirms that vehicle speed influences the identified parameters to some extent. Consequently, the results presented in the following subsections should be interpreted as reflecting the combined effects of speed, preprocessing, and model structure, rather than preprocessing alone.

### 6.4. ARX Identification Results

The objective of the ARX analysis is to evaluate the sensitivity of the ARX structure to the structured high-frequency disturbance and to investigate how preprocessing influences the identified dynamics, prediction performance, and dominant pole behavior. The ARX models were identified using the low-order structures selected in the previous subsection. Since the ARX formulation does not contain an explicit disturbance model, structured disturbances directly affect the deterministic part of the identified system and may therefore influence the estimated pole-zero structure. Table 3 summarizes the prediction performance obtained for the investigated preprocessing cases.

The ARX results show a clear dependence on preprocessing. Raw datasets generally produced the least favorable prediction metrics and less consistent dominant pole locations, suggesting that part of the structured disturbance was absorbed into the deterministic model. Notch filtering reduced the influence of the narrow-band disturbance around 10 Hz and provided a moderate improvement in most cases. Low-pass-filtered signals generally provided the most consistent overall performance, which yielded lower error measures. Although resampling further simplified some of the identified models, the resulting systems are not directly comparable to the original 30 Hz models because of the changed sampling frequency.

Table 4 presents the mean dominant pole locations of the identified second-order ARX models. Zeros are not reported separately, since their locations were found to be more sensitive to preprocessing and repetition-to-repetition variations, whereas the dominant poles provide a more compact and robust representation of the low-frequency dynamics relevant for the present analysis. The reported values were obtained by averaging the dominant poles identified from repeated measurements conducted under identical test conditions. Complex-conjugate poles are reported in the form a±bi, where ± denotes the corresponding conjugate pair.

The dominant pole locations reveal a strong dependence on preprocessing for the slalom measurements. Although the free-driving tests produced nearly identical dominant poles for all preprocessing methods, the raw and notch-filtered slalom datasets produced unstable dominant poles in the $30\;\frac{\mathrm{km}}{h}$ case. Low-pass filtering consistently shifted the dominant poles toward stable and dynamically plausible locations. Together with the prediction metrics, these results indicate that the ARX structure is sensitive to the structured disturbance around 10 Hz and tends to absorb part of the disturbance-related dynamics into the deterministic model.

Overall, the ARX results confirm that preprocessing plays a significant role in the identification process. Among the investigated approaches, low-pass filtering provides the most consistent improvements in both prediction performance and dominant pole behavior.

### 6.5. IV-ARX Identification Results

The IV-ARX analysis was included in order to assess the sensitivity of the identification results to possible closed-loop bias originating from the measurement configuration. In contrast to standard ARX estimation, the IV-ARX approach applies an instrumental-variable estimator, which is less sensitive to regressor-disturbance correlation, but may also become more sensitive to limited excitation and numerical conditioning.

Table 5 summarizes the prediction performance for the investigated preprocessing cases. Compared to the ARX results, the IV-ARX models exhibit substantially less consistent behavior. In some cases, such as the free-driving test at $65\;\frac{\mathrm{km}}{h}$ and the slalom tests at 50 and $70\;\frac{\mathrm{km}}{h}$, the raw, notch-filtered, and low-pass-filtered datasets yield comparable performance, with moderate improvements after preprocessing. However, in other cases, low-pass filtering and resampling lead to severe performance degradation, including strongly deteriorated fit values.

Table 6 presents the mean dominant pole locations of the identified second-order IV-ARX models. The pole results confirm that the IV-ARX estimates are substantially less robust than the corresponding ARX and ARMAX models. While some cases yield dominant poles in a plausible low-frequency region, several others produce unstable or clearly non-physical pole locations, especially after low-pass filtering or resampling. This behavior indicates that, under the present measurement conditions, the instrumental-variable estimator is more sensitive to limited excitation and estimation conditioning than the conventional ARX approach.

Overall, the IV-ARX results do not support the use of instrumental-variable estimation as a generally superior alternative for the present application. Nevertheless, they provide a useful benchmark despite its limited numerical robustness from a closed-loop identification perspective. In particular, the fact that the IV-ARX models remain acceptable in several of the raw and notch-filtered cases suggests that the main low-frequency vehicle dynamics can still be recovered under driver-generated excitation, while the observed instabilities highlight the practical limitations of instrumental-variable estimation when the available excitation is limited and the data contain structured disturbances.

### 6.6. ARMAX Identification Results

The ARMAX analysis investigates whether the explicit noise model can reduce the influence of the structured high-frequency disturbance. The ARMAX models were identified using the low-order structures selected in the previous subsection. In contrast to ARX models, the ARMAX formulation includes a moving-average disturbance model, allowing part of the structured disturbance to be represented separately from the deterministic vehicle dynamics. Consequently, the identified system dynamics are expected to be less sensitive to disturbance-related spectral components, potentially resulting in improved robustness and more physically meaningful model structures. Table 7 summarizes the prediction performance obtained for the investigated preprocessing cases.

Compared to the ARX models, the ARMAX structure exhibits a substantially lower sensitivity to preprocessing, and it also behaves considerably more consistently than the IV-ARX benchmark. For all driving scenarios, only minor differences can be observed between the raw, notch-filtered, and low-pass-filtered datasets. Although low-pass filtering consistently provides the best numerical performance, the improvements are considerably smaller than those observed for ARX models, which indicates that the explicit disturbance model already absorbs a significant portion of the structured disturbance. In contrast, resampled datasets generally degrade the identification results for slalom measurements.

Table 8 presents the mean dominant pole locations of the identified second-order ARMAX models. As in the ARX case, only the dominant poles are reported, because they provide the most robust summary of the identified low-frequency dynamics.

The dominant pole analysis confirms the conclusions drawn from the prediction metrics. Unlike the ARX and IV-ARX models, all dominant poles remain stable, and the variations between preprocessing methods are substantially smaller. These results indicate that the explicit disturbance model successfully captures a significant portion of the disturbance-related dynamics, reducing the need for aggressive preprocessing while still benefiting from moderate filtering.

Overall, the ARMAX results provide the most robust and physically consistent identification results among the investigated model classes. While low-pass filtering still yields a small but systematic improvement, the ARMAX structure already mitigates much of the sensitivity to the structured disturbance through its explicit disturbance model. Compared with the ARX models, the performance gain achieved by preprocessing is generally smaller for ARMAX. This suggests that explicit disturbance modeling already compensates for a substantial part of the structured disturbance.

## 7. Practical Guidelines for Disturbance Handling

The previous sections compared different preprocessing strategies and identification methods for handling structured disturbances in lateral vehicle dynamics identification. Rather than identifying a universally optimal solution, the results show that the appropriate strategy depends on both the characteristics of the measured data and the objective of the identification task.

When frequency-domain analysis reveals a narrow-band disturbance outside the frequency range of the relevant vehicle dynamics, targeted preprocessing is generally the preferred approach. In the investigated measurements, the dominant vehicle dynamics are concentrated below approximately 2–3 Hz, while the structured disturbance appears around 10 Hz. Consequently, notch filtering effectively suppresses the disturbance without affecting the relevant dynamics. If additional broadband high-frequency noise is present, low-pass filtering provides a suitable alternative by attenuating both the narrow-band disturbance and the remaining high-frequency components.

When preprocessing is not possible, or when preservation of the original measured signals is required, explicit disturbance modeling becomes advantageous. In this study, ARMAX models consistently achieved equal or better prediction performance than the corresponding ARX models while requiring only low-order deterministic dynamics. The additional noise model allows part of the structured disturbance to be represented separately from the vehicle dynamics, thereby reducing the tendency of the deterministic model to absorb non-physical dynamics.

The comparison with IV-ARX models further indicates that the main conclusions remain valid even when an estimator with reduced sensitivity to closed-loop bias is employed. Although the IV-ARX estimator does not necessarily improve the numerical prediction performance, it provides a useful reference for assessing the robustness of the identified dynamics under driver-in-the-loop operating conditions.

Increasing the deterministic model order alone is not recommended as the primary strategy for handling structured disturbances. While higher-order ARX models generally provide a slightly better numerical fit, the improvement becomes marginal beyond second-order models and may lead to unnecessarily complex model structures. In contrast, disturbance mitigation through preprocessing or explicit noise modeling produces more consistent improvements while preserving physically interpretable low-order vehicle dynamics.

The practical recommendations can be summarized as follows. Based on the obtained results, the following practical decision procedure is recommended:

1. Perform a frequency-domain analysis of the measured signals.
2. If no distinct high-frequency spectral component is observed, standard low-order ARX identification is generally sufficient.
3. If a narrow-band disturbance is detected outside the frequency range of the relevant vehicle dynamics and its frequency is known, apply targeted notch filtering prior to identification.
4. If broadband high-frequency noise is also present, apply low-pass filtering instead of increasing the deterministic model order.
5. If preprocessing cannot be applied, or if preserving the original measured signals is required, use an ARMAX model to account for structured disturbances through explicit noise modeling.
6. Use IV-ARX as a complementary benchmark when closed-loop bias caused by driver-in-the-loop measurements is suspected.

**Figure 13:**
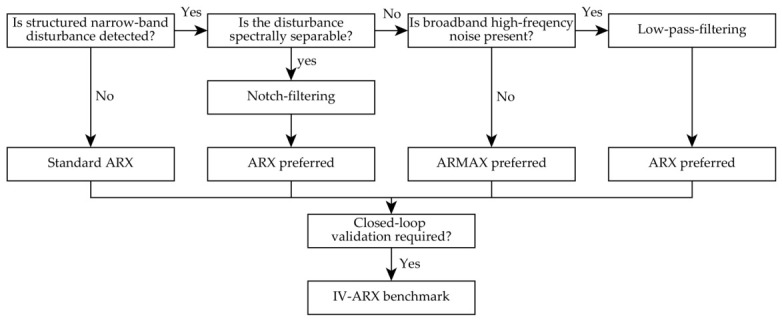
Recommended engineering workflow for selecting an appropriate disturbance-handling strategy based on the spectral characteristics of the measured signals.

The proposed workflow is summarized in Figure 13, which provides a concise engineering-oriented decision aid for selecting an appropriate disturbance-handling strategy in practical linear vehicle dynamics identification.

Resampling is intentionally omitted from the proposed workflow. Based on the obtained results, it should not be considered a primary disturbance-mitigation technique, but rather an implementation-oriented step that may be applied after appropriate anti-alias low-pass filtering when a reduced sampling frequency is required.

## 8. Discussion

The results consistently demonstrate that weak but persistent structured disturbances may influence linear black-box identification even when their energy is considerably lower than that of the dominant system dynamics. Although the investigated disturbance is located around 10 Hz, well above the frequency range containing the relevant lateral vehicle dynamics, it still increases the complexity required by deterministic model structures if it is not treated appropriately.

The comparison of the investigated identification approaches indicates that disturbance mitigation is generally more effective than increasing the deterministic model order. Higher-order ARX models provide only marginal improvements in prediction accuracy while introducing additional poles that are difficult to interpret from a physical viewpoint. In contrast, targeted preprocessing or explicit disturbance modeling produces similar or better prediction performance while preserving low-order models that remain consistent with the expected vehicle dynamics.

The results also highlight the different roles of the investigated model structures. When the disturbance is spectrally separated from the relevant dynamics, notch filtering offers the most selective solution by removing the narrow-band component without affecting the low-frequency vehicle response. Low-pass filtering provides a more general preprocessing strategy that additionally suppresses broadband measurement noise and therefore yields the most consistent overall identification results. If preprocessing is not feasible, ARMAX models successfully compensate for a large part of the disturbance through the explicit noise model, making them considerably less sensitive to structured disturbances than ARX models. The IV-ARX estimator does not improve the identification accuracy in the present application but supports the robustness of the main conclusions, which remain valid despite the possible closed-loop effects introduced by driver-in-the-loop measurements.

Although the study focuses on lateral vehicle dynamics, the proposed methodology may also be applicable to other control-oriented system identification problems in which the relevant system dynamics and structured disturbances occupy well-separated frequency ranges.

## 9. Conclusions

This paper investigated the influence of weak but persistent high-frequency structured disturbances on the linear identification of lateral vehicle dynamics using experimental driver-in-the-loop measurements. Frequency-domain analysis showed that the dominant vehicle dynamics are concentrated below approximately 2–3 Hz, while a narrow-band disturbance around 10 Hz is consistently present in the steering signal.

The comparison of ARX, IV-ARX and ARMAX models under different preprocessing conditions demonstrated that increasing the deterministic model order alone is generally not an effective way to handle such disturbances. Instead, selective preprocessing or explicit disturbance modeling provides more robust and physically interpretable models. Among the investigated approaches, notch filtering proved to be the preferred solution when the disturbance frequency is known and spectrally separated from the relevant dynamics, whereas low-pass filtering yielded the most consistent overall performance by simultaneously suppressing narrow-band disturbances and broadband high-frequency noise. When preprocessing cannot be applied, ARMAX models provide a practical alternative by representing part of the disturbance through the noise model.

The proposed engineering workflow offers a simple and practically applicable guideline for selecting disturbance-handling strategies based on the spectral characteristics of the measured signals. Future work will extend the proposed methodology to nonlinear vehicle models and more complex disturbance scenarios involving multiple structured frequency components.

## Figures and Tables

**Table 1:** Comparison of ARX, IV-ARX, and ARMAX model orders for the investigated driving scenarios using raw measurement data.

Order	ARX			IV-ARX			ARMAX		
	FPE	BIC	FIT	FPE	BIC	FIT	FPE	BIC	FIT
Free-driving at $50\;\frac{\mathrm{km}}{h}$									
1	$3.82\times 10^{-3}$	$-1.05\times 10^{4}$	96.41%	$7.54\times 10^{-3}$	$-7.88\times 10^{3}$	94.96%	$3.77\times 10^{-3}$	$-1.06\times 10^{4}$	96.43%
2	$3.21\times 10^{-3}$	$-1.12\times 10^{4}$	96.71%	$6.23\times 10^{-3}$	$-8.70\times 10^{3}$	95.45%	$2.60\times 10^{-3}$	$-1.20\times 10^{4}$	97.04%
3	$2.92\times 10^{-3}$	$-1.15\times 10^{4}$	96.87%	$2.11\times 10^{-2}$	$-6.21\times 10^{3}$	92.58%	$2.44\times 10^{-3}$	$-1.22\times 10^{4}$	97.14%
4	$2.62\times 10^{-3}$	$-1.19\times 10^{4}$	97.04%	$4.95\times 10^{-2}$	$-6.26\times 10^{2}$	87.15%	$2.41\times 10^{-3}$	$-1.22\times 10^{4}$	97.16%
Free-driving at $65\;\frac{\mathrm{km}}{h}$									
1	$7.51\times 10^{-3}$	$-5.69\times 10^{3}$	96.95%	$7.79\times 10^{-2}$	$-1.03\times 10^{3}$	91.35%	$7.49\times 10^{-3}$	$-5.69\times 10^{3}$	96.95%
2	$5.92\times 10^{-3}$	$-6.33\times 10^{3}$	97.30%	$1.20\times 10^{-2}$	$-4.37\times 10^{3}$	96.15%	$4.66\times 10^{-3}$	$-6.99\times 10^{3}$	97.60%
3	$5.20\times 10^{-3}$	$-6.67\times 10^{3}$	97.47%	$4.17\times 10^{-2}$	$-8.92\times 10^{2}$	92.83%	$4.64\times 10^{-3}$	$-6.99\times 10^{3}$	97.61%
4	$4.71\times 10^{-3}$	$-6.93\times 10^{3}$	97.59%	$6.11\times 10^{-2}$	$-1.02\times 10^{3}$	92.06%	$4.61\times 10^{-3}$	$-6.99\times 10^{3}$	97.62%
Slalom at $30\;\frac{\mathrm{km}}{h}$									
1	$7.45\times 10^{-3}$	$-1.83\times 10^{3}$	96.26%	$7.76\times 10^{-3}$	$-1.78\times 10^{3}$	96.16%	$7.22\times 10^{-3}$	$-1.85\times 10^{3}$	96.32%
2	$6.25\times 10^{-3}$	$-1.98\times 10^{3}$	96.60%	$1.94\times 10^{-1}$	$-1.79\times 10^{2}$	83.56%	$4.92\times 10^{-3}$	$-2.18\times 10^{3}$	96.99%
3	$5.34\times 10^{-3}$	$-2.10\times 10^{3}$	96.89%	$1.88\times 10^{-2}$	$-9.42\times 10^{2}$	94.00%	$4.50\times 10^{-3}$	$-2.25\times 10^{3}$	97.14%
4	$4.46\times 10^{-3}$	$-2.23\times 10^{3}$	97.15%	$2.73\times 10^{-2}$	$-1.01\times 10^{3}$	93.51%	$4.40\times 10^{-3}$	$-2.25\times 10^{3}$	97.18%
Slalom at $50\;\frac{\mathrm{km}}{h}$									
1	$9.25\times 10^{-2}$	$2.57\times 10^{2}$	92.39%	$2.32\times 10^{-1}$	$7.53\times 10^{2}$	87.96%	$5.60\times 10^{-2}$	$-1.43\times 10^{1}$	94.08%
2	$1.92\times 10^{-2}$	$-5.79\times 10^{2}$	96.54%	$2.23\times 10^{-2}$	$-4.99\times 10^{2}$	96.26%	$1.07\times 10^{-2}$	$-8.98\times 10^{2}$	97.41%
3	$1.32\times 10^{-2}$	$-7.69\times 10^{2}$	97.14%	$2.77\times 10^{-1}$	$5.59\times 10^{2}$	88.76%	$1.01\times 10^{-2}$	$-9.14\times 10^{2}$	97.50%
4	$1.01\times 10^{-2}$	$-9.04\times 10^{2}$	97.52%	$2.39\times 10^{-2}$	$-5.44\times 10^{2}$	96.31%	$9.81\times 10^{-3}$	$-9.17\times 10^{2}$	97.55%
Slalom at $70\;\frac{\mathrm{km}}{h}$									
1	$6.31\times 10^{-2}$	$3.40\times 10^{1}$	93.45%	$8.84\times 10^{-2}$	$1.84\times 10^{2}$	92.28%	$3.91\times 10^{-2}$	$-1.82\times 10^{2}$	94.83%
2	$1.82\times 10^{-2}$	$-5.15\times 10^{2}$	96.47%	$2.27\times 10^{-2}$	$-4.18\times 10^{2}$	96.07%	$9.64\times 10^{-3}$	$-8.13\times 10^{2}$	97.44%
3	$1.18\times 10^{-2}$	$-7.04\times 10^{2}$	97.18%	$1.13\times 10^{0}$	$5.78\times 10^{2}$	79.76%	$9.44\times 10^{-3}$	$-8.11\times 10^{2}$	97.49%
4	$9.27\times 10^{-3}$	$-8.04\times 10^{2}$	97.52%	$4.70\times 10^{-1}$	$3.94\times 10^{2}$	86.52%	$9.28\times 10^{-3}$	$-8.07\times 10^{2}$	97.53%

**Table 2:** Identified parameters of the second-order models obtained from raw measurements at different vehicle speeds (mean ± standard deviation of repeated measurements).

Parameter	Free-Driving		Slalom		
	$50\;\frac{\mathrm{km}}{h}$	$65\;\frac{\mathrm{km}}{h}$	$30\;\frac{\mathrm{km}}{h}$	$50\;\frac{\mathrm{km}}{h}$	$70\;\frac{\mathrm{km}}{h}$
ARX					
$a_{1}$	−0.6464±0.0135	−0.6436±0.0538	−1.2143±0.1605	−1.5584±0.0500	−1.2914±0.0694
$a_{2}$	−0.2972±0.0112	−0.3155±0.0767	0.1559±0.1304	0.5811±0.0391	0.4711±0.0962
$b_{1}$	1.1340±0.0638	1.4033±0.0440	0.1975±0.1305	0.3402±0.0607	0.6811±0.1132
$b_{2}$	−1.0884±0.0618	−1.3567±0.0712	−0.2306±0.1109	−0.3284±0.0505	−0.4560±0.1592
IV-ARX					
$a_{1}$	0.2673±0.5691	−1.7348±0.0264	−1.1775±0.4798	−1.8438±0.1045	−1.3022±1.0404
$a_{2}$	−1.2043±0.5159	0.7462±0.0298	0.2864±0.4028	0.8997±0.0915	0.7453±0.3647
$b_{1}$	2.0202±0.5581	0.3052±0.0309	0.5122±0.3208	0.1539±0.0986	0.4346±0.6407
$b_{2}$	−1.9696±0.5148	−0.2924±0.0350	−0.4763±0.2952	−0.1124±0.0870	0.1210±0.2227
ARMAX					
$a_{1}$	−1.0580±0.0395	−1.5592±0.0270	−1.6392±0.1402	−1.7752±0.0143	−1.7201±0.0250
$a_{2}$	0.0672±0.0381	0.5659±0.0280	0.6784±0.1615	0.8294±0.0123	0.7913±0.0155
$b_{1}$	0.8094±0.0459	0.5104±0.0249	0.2026±0.0323	0.2142±0.0184	0.2906±0.0189
$b_{2}$	−0.8023±0.0448	−0.5031±0.0262	−0.1910±0.0363	−0.1744±0.0145	−0.2069±0.0139
$c_{1}$	−0.6125±0.0310	−1.0100±0.0322	−0.7184±0.1164	−0.9062±0.0682	−0.9526±0.1289
$c_{2}$	−0.1097±0.0200	0.2228±0.0774	0.2138±0.0406	0.3632±0.0322	0.4344±0.0548

**Table 3:** Comparison of ARX identification results under different preprocessing conditions.

Preprocessing	MSE	FPE	BIC	FIT
Free-driving at $50\;\frac{\mathrm{km}}{h}$				
Raw	$3.20\times 10^{-3}$	$3.20\times 10^{-3}$	$-1.12\times 10^{4}$	96.71%
Notch-filtered	$2.92\times 10^{-3}$	$2.92\times 10^{-3}$	$-1.15\times 10^{4}$	96.86%
Low-pass-filt.	$2.82\times 10^{-3}$	$2.82\times 10^{-3}$	$-1.17\times 10^{4}$	96.92%
Resampled	$1.81\times 10^{-3}$	$1.82\times 10^{-3}$	$-4.44\times 10^{3}$	97.39%
Free-driving at $65\;\frac{\mathrm{km}}{h}$				
Raw	$5.90\times 10^{-3}$	$5.92\times 10^{-3}$	$-6.34\times 10^{3}$	97.30%
Notch-filtered	$5.02\times 10^{-3}$	$5.04\times 10^{-3}$	$-6.79\times 10^{3}$	97.50%
Low-pass-filt.	$4.69\times 10^{-3}$	$4.71\times 10^{-3}$	$-6.98\times 10^{3}$	97.59%
Resampled	$4.43\times 10^{-3}$	$4.47\times 10^{-3}$	$-2.37\times 10^{3}$	97.54%
Slalom at $30\;\frac{\mathrm{km}}{h}$				
Raw	$6.16\times 10^{-3}$	$6.22\times 10^{-3}$	$-1.99\times 10^{3}$	96.60%
Notch-filtered	$5.62\times 10^{-3}$	$5.67\times 10^{-3}$	$-2.09\times 10^{3}$	96.78%
Low-pass-filt.	$3.74\times 10^{-3}$	$3.78\times 10^{-3}$	$-2.41\times 10^{3}$	97.35%
Resampled	$1.44\times 10^{-2}$	$1.49\times 10^{-2}$	$-4.14\times 10^{2}$	94.85%
Slalom at $50\;\frac{\mathrm{km}}{h}$				
Raw	$1.79\times 10^{-2}$	$1.81\times 10^{-2}$	$-6.70\times 10^{2}$	96.60%
Notch-filtered	$1.55\times 10^{-2}$	$1.58\times 10^{-2}$	$-7.52\times 10^{2}$	96.83%
Low-pass-filt.	$9.92\times 10^{-3}$	$1.01\times 10^{-2}$	$-1.01\times 10^{3}$	97.47%
Resampled	$2.82\times 10^{-2}$	$2.94\times 10^{-2}$	$-1.32\times 10^{2}$	95.85%
Slalom at $70\;\frac{\mathrm{km}}{h}$				
Raw	$1.77\times 10^{-2}$	$1.81\times 10^{-2}$	$-5.28\times 10^{2}$	96.47%
Notch-filtered	$1.51\times 10^{-2}$	$1.54\times 10^{-2}$	$-6.02\times 10^{2}$	96.75%
Low-pass-filt.	$9.34\times 10^{-3}$	$9.50\times 10^{-3}$	$-8.27\times 10^{2}$	97.45%
Resampled	$2.50\times 10^{-2}$	$2.64\times 10^{-2}$	$-1.22\times 10^{2}$	95.98%

**Table 4:** Mean dominant ARX pole locations obtained from repeated measurements.

Measurement	Raw	Notch-Filtered	Low-Pass-Filtered	Resampled
Free-driving at $50\;\frac{\mathrm{km}}{h}$	0.9737	0.9570	0.9522	0.9592
Free-driving at $65\;\frac{\mathrm{km}}{h}$	0.9759	0.9686	0.9674	0.9151
Slalom at $30\;\frac{\mathrm{km}}{h}$	1.1441	1.0714	0.8809	0.8137±0.2245i
Slalom at $50\;\frac{\mathrm{km}}{h}$	0.9847±0.0272i	0.9411	0.9275	0.6899±0.4261i
Slalom at $70\;\frac{\mathrm{km}}{h}$	0.7700±0.3877i	0.6457±0.2024i	0.9137	0.6114±0.3830i

**Table 5:** Comparison of IV-ARX identification results under different preprocessing conditions.

Preprocessing	MSE	FPE	BIC	FIT
Free-driving at $50\;\frac{\mathrm{km}}{h}$				
Raw	$6.21\times 10^{-3}$	$6.23\times 10^{-3}$	$-8.70\times 10^{3}$	95.45%
Notch-filtered	$6.18\times 10^{-3}$	$6.20\times 10^{-3}$	$-8.93\times 10^{3}$	95.51%
Low-pass-filt.	$9.63\times 10^{1}$	$9.66\times 10^{1}$	$1.69\times 10^{4}$	−312.38%
Resampled	$6.44\times 10^{-1}$	$6.50\times 10^{-1}$	$4.47\times 10^{2}$	62.96%
Free-driving at $65\;\frac{\mathrm{km}}{h}$				
Raw	$1.19\times 10^{-2}$	$1.20\times 10^{-2}$	$-4.37\times 10^{3}$	96.15%
Notch-filtered	$1.17\times 10^{-2}$	$1.17\times 10^{-2}$	$-4.43\times 10^{3}$	96.20%
Low-pass-filt.	$7.63\times 10^{-3}$	$7.67\times 10^{-3}$	$-5.62\times 10^{3}$	96.93%
Resampled	$4.67\times 10^{-3}$	$4.73\times 10^{-3}$	$-2.31\times 10^{3}$	97.47%
Slalom at $30\;\frac{\mathrm{km}}{h}$				
Raw	$1.92\times 10^{-1}$	$1.94\times 10^{-1}$	$-1.79\times 10^{2}$	83.56%
Notch-filtered	$9.59\times 10^{-3}$	$9.75\times 10^{-3}$	$-1.71\times 10^{3}$	95.89%
Low-pass-filt.	$5.34\times 10^{-1}$	$5.45\times 10^{-1}$	$6.53\times 10^{2}$	75.92%
Resampled	$1.74\times 10^{1}$	$1.80\times 10^{1}$	$8.01\times 10^{2}$	5.04%
Slalom at $50\;\frac{\mathrm{km}}{h}$				
Raw	$2.19\times 10^{-2}$	$2.23\times 10^{-2}$	$-4.99\times 10^{2}$	96.26%
Notch-filtered	$1.84\times 10^{-2}$	$1.88\times 10^{-2}$	$-5.91\times 10^{2}$	96.57%
Low-pass-filt.	$1.49\times 10^{-2}$	$1.52\times 10^{-2}$	$-7.04\times 10^{2}$	96.92%
Resampled	$5.44\times 10^{-1}$	$5.81\times 10^{-1}$	$2.01\times 10^{2}$	84.97%
Slalom at $70\;\frac{\mathrm{km}}{h}$				
Raw	$2.21\times 10^{-2}$	$2.27\times 10^{-2}$	$-4.18\times 10^{2}$	96.07%
Notch-filtered	$3.36\times 10^{-2}$	$3.45\times 10^{-2}$	$-2.78\times 10^{2}$	95.27%
Low-pass-filt.	$2.16\times 10^{-2}$	$2.22\times 10^{-2}$	$-4.59\times 10^{2}$	96.20%
Resampled	$1.47\times 10^{-1}$	$1.59\times 10^{-1}$	$6.23\times 10^{1}$	91.69%

**Table 6:** Mean dominant IV-ARX pole locations obtained from repeated measurements.

Measurement	Raw	Notch-Filtered	Low-Pass-Filtered	Resampled
Free-driving at $50\;\frac{\mathrm{km}}{h}$	0.9352±0.0465i	−0.3226	124.6183	15.4485
Free-driving at $65\;\frac{\mathrm{km}}{h}$	0.9465	0.9387	0.6413	0.7856
Slalom at $30\;\frac{\mathrm{km}}{h}$	−1.8183	0.8230±0.0218i	9.4268	31.6584
Slalom at $50\;\frac{\mathrm{km}}{h}$	1.0058±0.2124i	0.9219±0.2200i	1.1077	3.0103±0.2826i
Slalom at $70\;\frac{\mathrm{km}}{h}$	1.0800±0.1957i	0.6511±0.3159i	1.2960	0.2685±0.8494i

**Table 7:** Comparison of ARMAX identification results under different preprocessing conditions.

Preprocessing	MSE	FPE	BIC	FIT
Free-driving at $50\;\frac{\mathrm{km}}{h}$				
Raw	$2.58\times 10^{-3}$	$2.59\times 10^{-3}$	$-1.20\times 10^{4}$	97.05%
Notch-filtered	$2.50\times 10^{-3}$	$2.51\times 10^{-3}$	$-1.21\times 10^{4}$	97.09%
Low-pass-filt.	$2.47\times 10^{-3}$	$2.47\times 10^{-3}$	$-1.22\times 10^{4}$	97.11%
Resampled	$1.29\times 10^{-3}$	$1.30\times 10^{-3}$	$-4.88\times 10^{3}$	97.79%
Free-driving at $65\;\frac{\mathrm{km}}{h}$				
Raw	$4.63\times 10^{-3}$	$4.65\times 10^{-3}$	$-7.01\times 10^{3}$	97.60%
Notch-filtered	$4.59\times 10^{-3}$	$4.60\times 10^{-3}$	$-7.04\times 10^{3}$	97.62%
Low-pass-filt.	$4.59\times 10^{-3}$	$4.60\times 10^{-3}$	$-7.04\times 10^{3}$	97.62%
Resampled	$3.07\times 10^{-3}$	$3.10\times 10^{-3}$	$-2.71\times 10^{3}$	97.94%
Slalom at $30\;\frac{\mathrm{km}}{h}$				
Raw	$4.83\times 10^{-3}$	$4.88\times 10^{-3}$	$-2.20\times 10^{3}$	97.00%
Notch-filtered	$4.49\times 10^{-3}$	$4.53\times 10^{-3}$	$-2.26\times 10^{3}$	97.11%
Low-pass-filt.	$3.58\times 10^{-3}$	$3.62\times 10^{-3}$	$-2.44\times 10^{3}$	97.40%
Resampled	$8.40\times 10^{-3}$	$8.66\times 10^{-3}$	$-5.67\times 10^{2}$	96.05%
Slalom at $50\;\frac{\mathrm{km}}{h}$				
Raw	$1.00\times 10^{-2}$	$1.02\times 10^{-2}$	$-1.01\times 10^{3}$	97.46%
Notch-filtered	$9.56\times 10^{-3}$	$9.69\times 10^{-3}$	$-1.04\times 10^{3}$	97.51%
Low-pass-filt.	$9.16\times 10^{-3}$	$9.28\times 10^{-3}$	$-1.06\times 10^{3}$	97.57%
Resampled	$1.38\times 10^{-2}$	$1.44\times 10^{-2}$	$-2.67\times 10^{2}$	97.09%
Slalom at $70\;\frac{\mathrm{km}}{h}$				
Raw	$9.08\times 10^{-3}$	$9.24\times 10^{-3}$	$-8.39\times 10^{2}$	97.48%
Notch-filtered	$8.78\times 10^{-3}$	$8.93\times 10^{-3}$	$-8.55\times 10^{2}$	97.53%
Low-pass-filt.	$8.34\times 10^{-3}$	$8.49\times 10^{-3}$	$-8.77\times 10^{2}$	97.59%
Resampled	$1.41\times 10^{-2}$	$1.49\times 10^{-2}$	$-2.12\times 10^{2}$	97.00%

**Table 8:** Mean dominant ARMAX pole locations obtained from repeated measurements.

Measurement	Raw	Notch-Filtered	Low-Pass-Filtered	Resampled
Free-driving at $50\;\frac{\mathrm{km}}{h}$	0.9903	0.9901	0.9905	0.9689
Free-driving at $65\;\frac{\mathrm{km}}{h}$	0.9828	0.9842	0.9792	0.9613
Slalom at $30\;\frac{\mathrm{km}}{h}$	0.9504±0.0880i	0.8964±0.1159i	0.8852	0.8625
Slalom at $50\;\frac{\mathrm{km}}{h}$	0.9322±0.2141i	0.8876±0.2034i	0.8375±0.0941i	0.5595±0.2312i
Slalom at $70\;\frac{\mathrm{km}}{h}$	0.8986±0.2512i	0.8601±0.2262i	0.8061±0.0699i	0.5279±0.4500i

## Data Availability

The raw measurement data supporting the findings of this study are publicly available at: https://github.com/istegy/Lexus_measurements (accessed on 8 June 2026).

## Abbreviations

The following abbreviations are used in this manuscript:

APSD	Auto power spectral density
ARX	Autoregressive with exogenous input
ARMAX	Autoregressive moving average with exogenous input
BIC	Bayesian information criterion
FPE	Final prediction error
IV-ARX	Instrumental-variable ARX
MSE	Mean squared error

## References

[1] Ljung L. (1999). System Identification: Theory for the User.

[2] Istenes G., Ignéczi G.F., Józsa D., Pup D., Bokor J. (2025). Spectral Analysis of the Lateral Dynamics of Road Vehicles. Eng. Proc..

[3] Rajamani R. (2011). Vehicle Dynamics and Control.

[4] Gillespie T. (2021). Fundamentals of Vehicle Dynamics.

[5] Jang J.H., Han C.S. (1997). The sensitivity analysis of yaw rate for a front wheel steering vehicle: In the frequency domain. KSME Int. J..

[6] Huang H.H., Tsai M.J. (2019). Vehicle Cornering Performance Evaluation and Enhancement Based on CAE and Experimental Analyses. Appl. Sci..

[7] Berntorp K., Di Cairano S. (2018). Offset and noise estimation of automotive-grade sensors using adaptive particle filtering. Proceedings of the 2018 Annual American Control Conference (ACC).

[8] Pietruch M., Wetula A., Mlyniec A. (2022). Influence of the accuracy and CAN frame period of the steering wheel angle sensor (SAS) on the trajectory of a steer-by-wire-equipped car. IEEE Access.

[9] Wang X. (2010). Vehicle Noise and Vibration Refinement.

[10] Navet N., Simonot-Lion F. (2009). Automotive Embedded Systems Handbook.

[11] Diversi R., Guidorzi R., Soverini U. (2010). Identification of ARX and ARARX Models in the Presence of Input and Output Noises. Eur. J. Control.

[12] Yu C., You K., Xie L. (2016). Quantized identification of ARMA systems with colored measurement noise. Automatica.

[13] Escobar J., Poznyak A. (2022). Robust Parametric Identification for ARMAX Models with Non-Gaussian and Coloured Noise: A Survey. Mathematics.

[14] Sun B., Teng Z., Hu Q., Lin H., Tang S. (2020). Periodic Noise Rejection of Checkweigher Based on Digital Multiple Notch Filter. IEEE Sens. J..

[15] El Gebali A., Landry R.J. (2022). Single and Multiple Continuous-Wave Interference Suppression Using Adaptive IIR Notch Filters Based on Direct-Form Structure in a QPSK Communication System. Appl. Sci..

[16] Hexagon Autonomy & Positioning (2024). Lexus RX450h PACMod 3.0 System Datasheet. https://autonomoustuff.com/-/media/Images/Hexagon/Hexagon-Core/autonomousstuff/pdf/as-lexus-pacmod-3-datasheet.ashx.

[17] Bonci A., Brunella F., Colletta M., Biase A.D., Dragoni A.F., Libofsha A. (2026). ROS 2-Based Architecture for Autonomous Driving Systems: Design and Implementation. Sensors.

[18] Johansson K.H., Törngren M., Nielsen L., Hristu-Varsakelis D., Levine W.S. (2005). Vehicle Applications of Controller Area Network. Handbook of Networked and Embedded Control Systems.

[19] Buscemi A., Turcanu I., Castignani G., Panchenko A., Engel T., Shin K.G. (2023). A Survey on Controller Area Network Reverse Engineering. IEEE Commun. Surv. Tutor..

[20] Forssell U., Ljung L. (1999). Closed-loop identification revisited. Automatica.

[21] Van den Hof P. (1998). Closed-loop issues in system identification. Annu. Rev. Control.

[22] Burnham K.P., Anderson D.R. (2002). Model Selection and Multimodel Inference.

